# Early recurrence of bladder cancer in the colon after robot‐assisted radical cystectomy: Disappearance following dose‐dense methotrexate, vinblastine, doxorubicin and cisplatin treatment

**DOI:** 10.1002/iju5.12370

**Published:** 2021-09-08

**Authors:** Michio Noda, Masaki Nakamura, Taketo Kawai, Yusuke Sato, Yuta Yamada, Yoshiyuki Akiyama, Daisuke Yamada, Motofumi Suzuki, Haruki Kume

**Affiliations:** ^1^ Department of Urology Mitsui Memorial Hospital Japan; ^2^ Department of Urology Graduate School of Medicine The University of Tokyo Japan; ^3^ Department of Urology NTT Medical Center Japan; ^4^ Department of Urology Tokyo Metropolitan Bokutoh Hospital Tokyo Japan

**Keywords:** and cisplatin, atypical recurrence, descending colon, dose‐dense methotrexate, doxorubicin, robot‐assisted radical cystectomy, vinblastine

## Abstract

**Introduction:**

The popularity of robot‐assisted radical cystectomy over open radical cystectomy has been increasing because the former, a minimally invasive surgery, contributes to earlier recovery and shorter hospitalization. However, atypical recurrences may be more frequent after robot‐assisted radical cystectomy than after open radical cystectomy. We report a case of an atypical early recurrence of bladder cancer including the descending colon.

**Case presentation:**

A 70‐year‐old Japanese man underwent robot‐assisted radical cystectomy for muscle‐invasive bladder cancer. Four months later, he was hospitalized for severe anemia (hemoglobin, 5.1 g/dL). Colonoscopy revealed a 4‐cm submucosal oozing tumor in the descending colon. Computed tomography revealed multiple recurrent lesions including recurrence in the descending colon, all of which disappeared completely after chemotherapy with six cycles of dose‐dense methotrexate, vinblastine, doxorubicin, and cisplatin.

**Conclusion:**

We encountered a rare case of an atypical recurrence of bladder cancer in the colon after robot‐assisted radical cystectomy.

**Abbreviations & Acronyms**
CR: Complete response
CT: computed tomography
dd‐MVAC: dose‐dense methotrexate, vinblastine, doxorubicin and cisplatin
ORC: open radical cystectomy
RARC: robot‐assisted radical cystectomy

**Keynote message**

We report a case of an atypical recurrence of bladder cancer in the descending colon after robot‐assisted radical cystectomy. Although there are several case reports of atypical recurrence after robot‐assisted radical cystectomy, recurrence in the colon has been rarely reported.

## Introduction

Since first being reported in 2003, RARC has been presented as a potential alternative surgical procedure to ORC with the advantages of reduced blood loss, earlier postoperative recovery, and a shorter hospitaization.^1^ However, some studies have raised concerns that atypical recurrences could be more frequent after RARC.^2^

Here, we report a case of an atypical early recurrence of bladder cancer in the descending colon after RARC.

## Case presentation

**Fig. 1:**
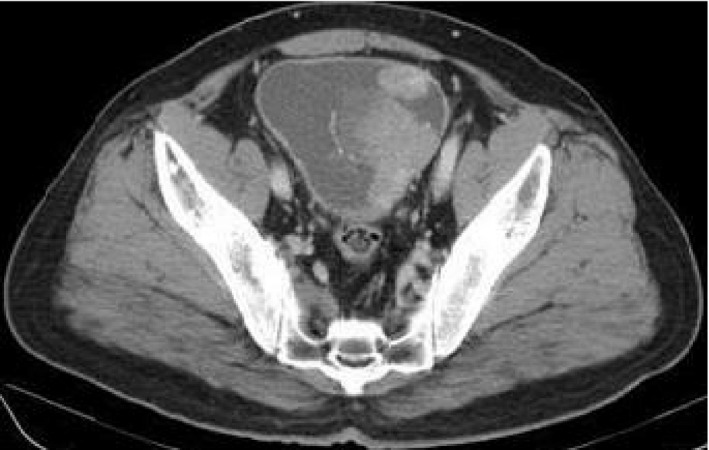
CT scan findings of the muscle‐invasive bladder cancer before RARC.

A 70‐year‐old Japanese man was admitted to our hospital with a chief complaint of dysuria. Abdominal ultrasonography revealed a large bladder tumor, and cystoscopy revealed a nodular tumor filling the left half of the urinary bladder. CT revealed a cT2bN0M0 bladder cancer (Fig. 1).

He underwent transurethral resection of the bladder tumor and was diagnosed with high‐grade pT2 urothelial carcinoma with glandular differentiation. Left hydronephrosis developed 2 weeks later due to obstruction of the lower part of the left ureter. We immediately planned radical cystectomy following left percutaneous nephrostomy without neoadjuvant chemotherapy. He underwent RARC with urethral resection with an intracorporeal ileal conduit urinary diversion. The extra fourth arm was set on the left side of the patient, and 12 and 5 mm ports for bed‐side assistant were set on the right side of the patient. The lymphadenectomy was performed as a level of extended template including the common iliac, external iliac, internal iliac, and presacral lymph nodes. In an ileal conduit procedure, an approximately 25 cm segment of ileum was isolated and the distal ends of both ureters were anastomosed to the ileum by the Bricker technique. The total operative time and console time were 571 and 476 min, respectively. The estimate blood loss was 570 mL. Insufflation pressure was 10 mmHg. Histopathological examination revealed high‐grade pT2b urothelial carcinoma with glandular differentiation, INFβ, lymphatic invasion (+), vascular invasion (−), resected margin (−), and pN0 (0/55). The postoperative course was uneventful, and he was discharged approximately 1 month after RARC.

**Fig. 2:**
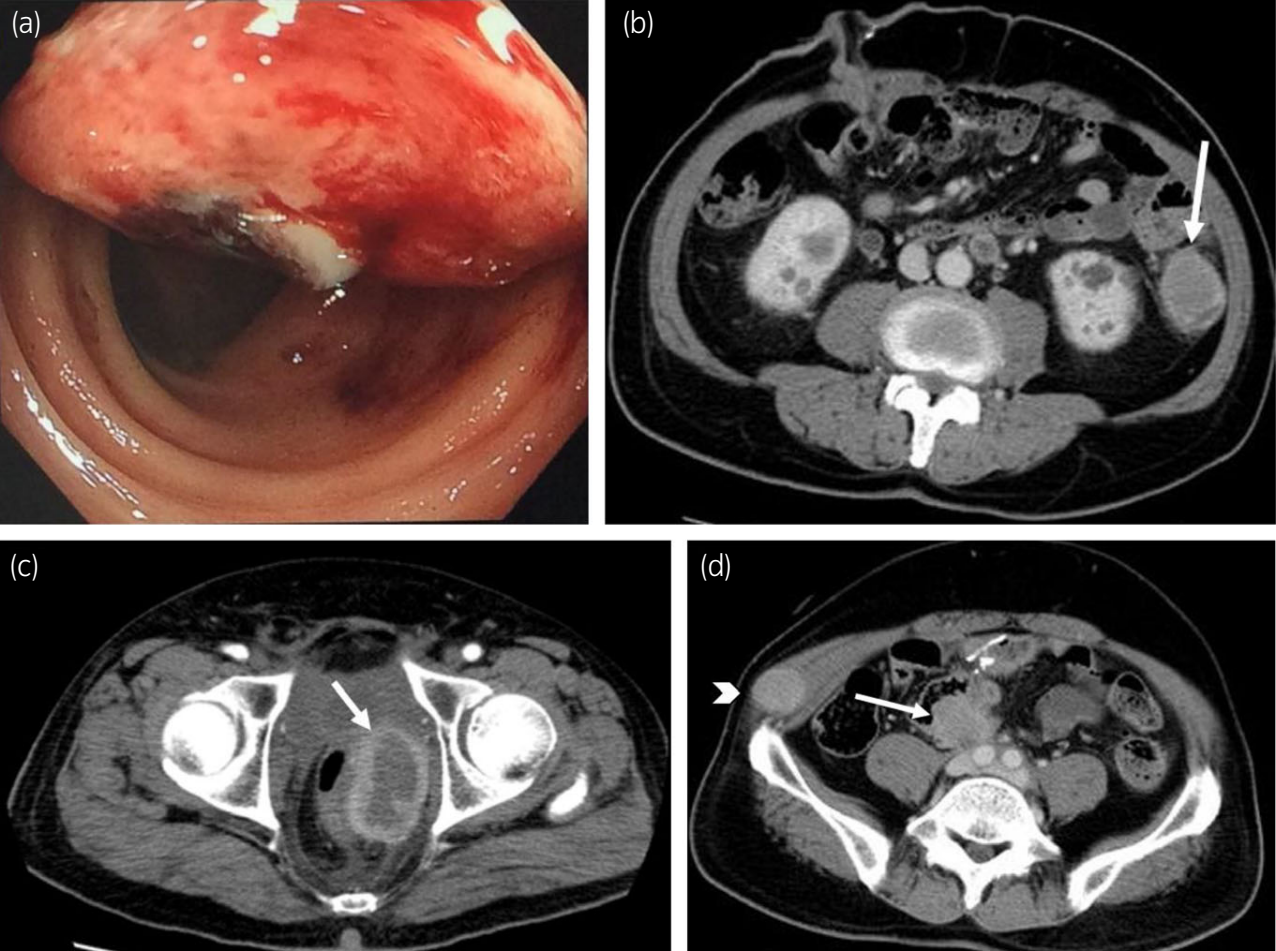
Multiple recurrences of bladder cancer after RARC; (a) colonoscopic findings showing an oozing submucosal tumor in the descending colon, (b–d) CT scan findings: (b) a 4‐cm intraperitoneal tumor in the descending colon (arrow), (c) a huge local recurrent mass in the pelvic floor (arrow) (d) para‐aortic lymph nodes (arrow) and nodules in the oblique abdominal muscles (arrowhead).

Four months later, he was admitted to our hospital emergently for shortness of breath; his hemoglobin level fell to 5.1 g/dL. Gastrointestinal bleeding was suspected, and he underwent colonoscopy, which showed a 4‐cm submucosal oozing tumor in the descending colon (Fig. 2a). Tumor biopsy was not performed due to the risk of bleeding.

Enhanced CT revealed intraperitoneal recurrence in the descending colon (Fig. 2b), massive local recurrent mass in the pelvic floor (Fig. 2c), swelling of the pelvic and para‐aortic lymph nodes, and nodules in the oblique abdominal muscles (Fig. 2d). Additionally, right hydronephrosis and hydroureter developed due to right ureteral obstruction by disseminated peritoneal metastasis.

**Fig. 3:**
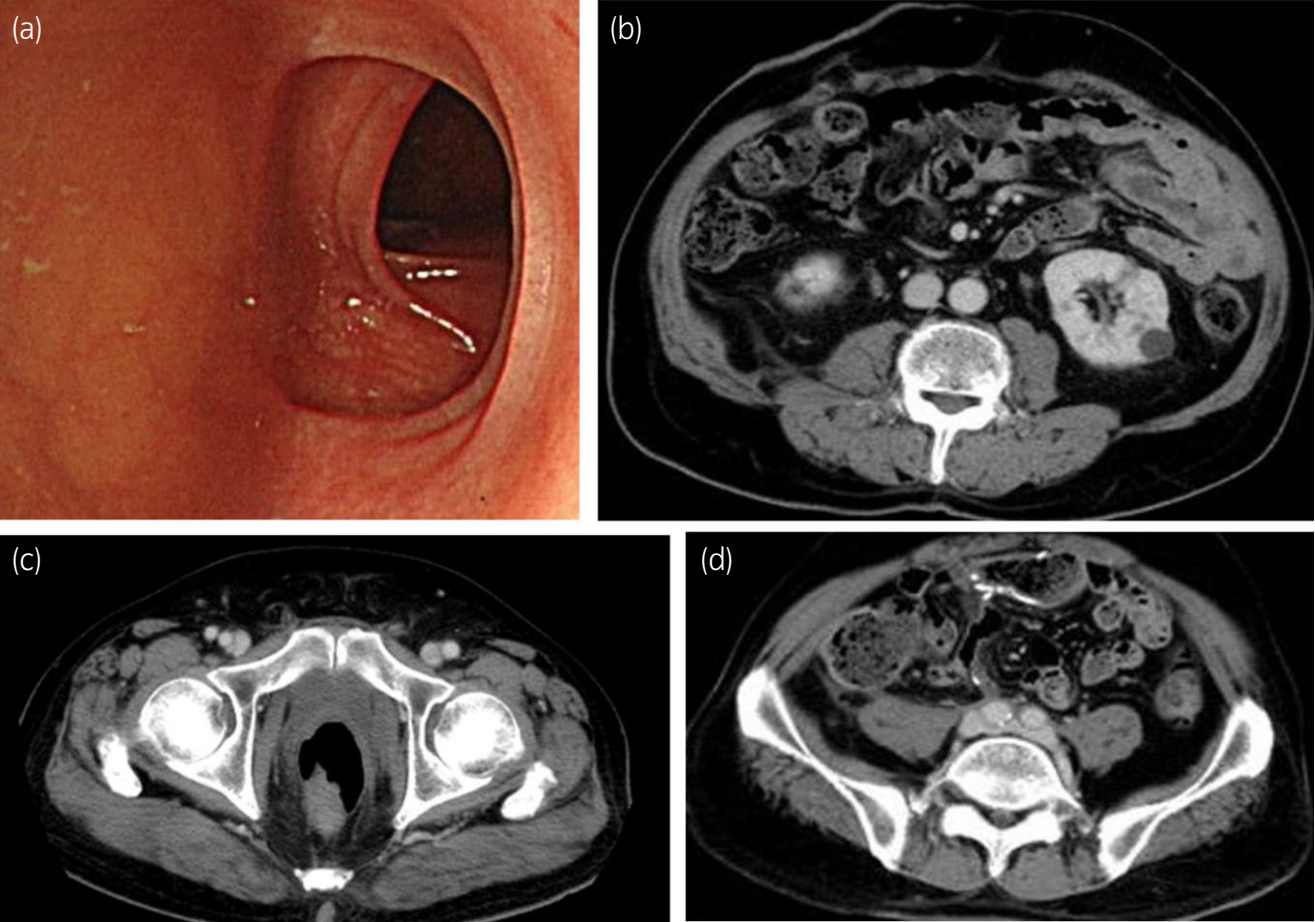
Complete response after dd‐MVAC treatment. Colonoscopic findings in the descending colon (a) and CT scan findings (b–d).

Five months after RARC, chemotherapy was initiated with dd‐MVAC, which consisted of methotrexate (30 mg/m^2^), vinblastine (3 mg/m^2^), doxorubicin (30 mg/m^2^) and cisplatin (70 mg/m^2^) every 2 weeks. He tolerated six cycles of the treatments without severe adverse events. CR according to the RECIST v1.1 was achieved (Fig. 3a–d). He has been followed for 1.5 years since RARC without evidence of recurrence.

## Discussion

The oncological and functional results of RARC and ORC are similar.^3^ With regard to 2‐year progression‐free survival, RARC is not inferior to ORC.^4^ Considering the advantages of RARC such as reduced blood loss and earlier postoperative recovery, RARC has been increasingly preferred over ORC in recent years.

Recurrences are of concern after radical cystectomy. Parekh *et al*. reported in the RAZOR trial that there were 33 of 150 (22%) and 35 of 152 (23%) distant metastases and 6 of 150 (4%) and 4 of 152 (3%) local recurrences in the RARC and ORC groups, respectively.^4^ Faraj *et al*. compared 203 RARC cases and 278 ORC cases with a median follow up of 66 months at Mayo Clinic and reported that there were 29 of 203 (14.3%) and 53 of 278 (19.1%) distant metastases and 12 of 203 (5.9%) and 19 of 278 (6.8%) local recurrences in the RARC and ORC groups, respectively.^5^ The most common sites of distant metastases were lungs, liver, bone, and extra pelvic lymph nodes in these two studies and these results was consistent with the metastatic patterns in autopsy studies.^6^ In these two studies, the metastasis to the intestine was observed in one case in the RARC group in the RAZOR trial.^4^

Although recent studies have shown similar rates of atypical recurrence after ORC and RARC,^5^, ^7^ there are substantial number of reports on atypical recurrences after RARC including peritoneal carcinomatosis and metastasis to extra‐pelvic lymph nodes and port sites.^2^, ^4^, ^5^, ^7^ The following three factors, including CO_2_ gas insufflation, surgical technique, and tumor biology are considered to relate to the occurrence of atypical recurrences.^8^ CO_2_ gas insufflation is essential in robot‐assisted urological surgeries. Experimental evidence suggests that pneumoperitoneum may increase the risk of intraperitoneal dissemination of cancer cells.^9^ One early study, comparing 263 cases of RARC to 120 cases of ORC at a single institution, showed that metastases to extra‐pelvic lymph nodes and peritoneal carcinomatosis were more frequent in patients after RARC than after ORC within 2 years after surgery.^2^ In the present case, we took precautionary measures, such as the immediate placing of the removed bladder into a surgical bag and immediate clipping of the Foley catheter after the dissection of the urethral wall, to prevent spreading malignant cells into the surgical field. Therefore, we assume that the high tumor burden and CO_2_ gas insufflation could be the reasons of atypical recurrence in our case.

In this case, fortunately, we observed an excellent clinical and radiologic response to dd‐MVAC chemotherapy, with atypical recurrences such as intraperitoneal recurrence in the descending colon disappearing completely. dd‐MVAC chemotherapy is known to be more effective than classic MVAC, which has similar effectiveness to GC treatment,^10^ for advanced urothelial tract tumors.^11^, ^12^ Sternberg *et al*. reported that dd‐MVAC treatment achieved a CR rate of 21% and a 5‐year survival rate of 21.8%.^12^ Although There are no randomized controlled trials directly comparing the effectiveness of dd‐MVAC and GC for advanced urothelial tract tumors, VESPER trial showed that dd‐MVAC provided a higher local control rate (complete pathological response, tumor downstaging, or organ confined) than GC in the neoadjuvant setting for muscle‐invasive bladder cacer.^13^ We expect that a CR will be achieved over the long term in the present case.

## Conclusion

We report herein a case of atypical early recurrence of bladder cancer after RARC with multiple lesions including recurrence in the descending colon, local recurrence in the pelvic floor, and metastases to the extra‐pelvic lymph nodes and a port site, which disappeared completely after six cycles of dd‐MVAC treatment. This is the first report of an earlyrecurrence in the descending colon after RARC.

## Author contribution

Michio Noda: writing‐original draft. Masaki Nakamura: writing‐original draft, writing‐review & editing, supervision. Taketo Kawai: writing‐review & editing, supervision. Yusuke Sato: writing‐review & editing. Yuta Yamada: writing‐review & editing. Yoshiyuki Akiyama: writing‐review & editing. Daisuke Yamada: writing‐review & editing. Motofumi Suzuki: writing‐review & editing. Haruki Kume: writing‐review & editing, supervision.

## Conflict of interest

None of the contributing authors have any conflict of interest, including specific financial interests or relationships and affiliations relevant to the subject matter or materials discussed in the manuscript.

## Approval of the research protocol by an Institutional Reviewer Board

Not applicable.

## Informed consent

All human subjects provided written informed consent with guarantees of confidentiality.

## Registry and the Registration No. of the study/trial

Not applicable.
